# Methemoglobin determination by multi-component analysis in coho salmon (*Oncorhynchus kisutch*) possessing unstable hemoglobin

**DOI:** 10.1016/j.mex.2020.100836

**Published:** 2020-02-22

**Authors:** Stephanie Blair, Clyde Barlow, Erin Martin, Ruth Schumaker, Jenifer McIntyre

**Affiliations:** a Department of Environmental Studies, The Evergreen State College, 2700 Evergreen Pkwy, Olympia, WA 98505, USA; b School of the Environment, Washington State University, Puyallup Research & Extension Center, 2606 Pioneer Ave, Puyallup, WA 98371, USA

**Keywords:** Methemoglobin, Coho salmon, Fish hemoglobin, Unstable hemoglobin, Hemichrome, Multi-component analysis

## Abstract

Hemoglobin derivatives are often quantified in blood to establish cardio-respiratory status and possible causes of impaired oxygen transport. The derivative known as methemoglobin results from oxidation of hemoglobin and is pathologically relevant because it cannot transport oxygen. In species and individuals possessing unstable methemoglobin, methemoglobin formation leads to rapid hemichrome formation and precipitation. Oxidizing reagents in standard methemoglobin analysis techniques therefore prevent accurate quantification of hemoglobin oxidative degradation products in species possessing unstable hemoglobin. In this study, we demonstrated that individual coho salmon (*Oncorhynchus kisutch*) possess unstable methemoglobin. Because molar absorptivities of coho methemoglobin, hemichrome and carboxyhemoglobin were significantly different from humans, the use of previous standard methods leads to an overestimation of methemoglobin in coho. Spontaneous conversion of methemoglobin to hemichrome was also demonstrated in Chinook (*O. tshawytscha*), pink (*O. gorbuscha*) and chum salmon (*O. keta*), but not steelhead (*O. mykiss*), indicating there may be a frequent need to account for unstable hemoglobin when quantifying methemoglobin in salmonids.

• Our method builds upon multi-component analysis (MCA) by using a multivariate modeling technique to derive the coho-specific molar absorptivities of major hemoglobin derivatives
• This approach fills a current need for the accurate quantification of methemoglobin in fishes possessing unstable hemoglobin

## Graphical abstract

**Figure:**
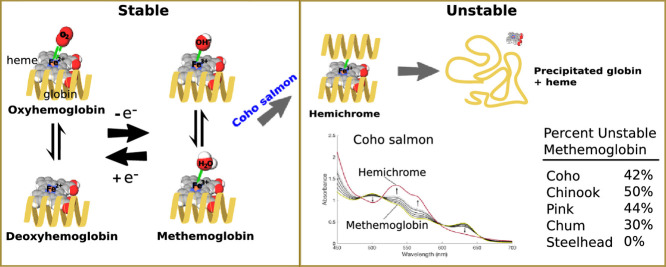

**Table:** Specification Table

Subject area:	*Environmental Science*
More specific subject area:	Hemoglobin quantification
Method name:	Coho (*Oncorhynchus kisutch*) hemoglobin multi-component analysis
Name and reference of original method:	Zijlstra, W. G., Buursma, A., & van Assendelft, O. W. (Eds.). (2000). *Visible and near infrared absorption spectra of human and animal hemoglobin: determination and application*. VSP.
Resource availability:	N/A

## Method details

Coho salmon (*Oncorhynchus kisutch*) are an ecologically, economically and culturally important species of anadromous Pacific salmon uniquely susceptible to acute mortality in response to urban stormwater runoff exposure [1]. While investigating whether methemoglobin is involved in this mortality phenomenon, we observed a precipitation interference after the addition of potassium ferricyanide to coho hemoglobin solutions, suggesting the formation of hemichromes from unstable methemoglobins. Because precipitation interfered with methemoglobin quantification using standard methods, we adapted and validated a MCA approach to methemoglobin determination in coho salmon following Zijlstra et al. [2]. We have determined and report here the molar absorptivities of major hemoglobin derivatives in coho salmon (Supplementary Table 1). Since coho methemoglobin could not be isolated to measure the pure absorbance spectra, the molar absorptivities of coho methemoglobin were obtained using multivariate statistics [3] based on the pseudo-first order reaction of the conversion of methemoglobin to hemichrome [4]. Due to the major influence of pH, molar absorptivities of coho methemoglobin from pH 8.0 to 6.8 are provided to meet a variety of assay conditions (Supplementary Tables 1 and 2).

The current method was developed to investigate methemoglobin formation in coho but may be applied more broadly as a valid approach for fish hemoglobin quantification. In these methods, we describe the relatively straightforward approach to quantifying coho methemoglobin using reported reference spectra and details for obtaining species-specific hemoglobin absorptivities in other fish species possessing unstable methemoglobins.

### Fish blood collection

For determination of coho hemoglobin molar absorptivities at pH 8.0, prespawn adult coho were netted from an artificial channel at the Quilcene National Fish Hatchery in Quilcene, WA, USA, during the month of August. Blood was non-lethally collected from five individual coho along the caudal vein, using 4-mL lithium heparin-lined vacutainers (Becton Dickinson, VWR) and 21-gauge/1.25-inch collection needles (Becton Dickinson Eclipse, VWR). Blood samples were stored on ice and immediately transported to the laboratory, where they were stored at 1–4 °C before preparing hemoglobin stock solutions the same day.

Blood samples for between-species comparisons of unstable methemoglobins and coho MCA methods validation were collected from prespawn adults at local hatcheries during routine artificial spawning events, immediately following euthanization by blow to the head. Species and sampling events included: chinook salmon (*O. tshawytscha*) returning to the George Adams National Fish Hatchery in Shelton, WA, USA during the month of August; pink salmon (*O. gorbuscha*) from the Hoodsport Hatchery in Hoodsport, WA, USA during the month of September; chum salmon (*O. keta*) from the Hoodsport Hatchery in Hoodsport, WA, USA during the month of November; coho from the Skookumchuck Hatchery in Tenino, WA, USA in the month of February; and steelhead (*O. mykiss*) from the Skookumchuck Hatchery in Tenino, WA, USA in the month of February.

For determination of coho methemoglobin molar absorptivities at pH 7.8 to pH 6.8, five hatchery origin juvenile coho were obtained from rearing tanks at Washington State University Puyallup Research and Extension Center in Puyallup, WA, USA. Blood was collected as above immediately after percussion stunning by blow to the head. After blood collection, fishes were euthanized by cervical transection. Blood samples were stored on ice until preparing hemoglobin stock solutions the same day.

### Preparation of hemoglobin stock solutions

In order to determine the molar absorptivities of coho hemoglobin derivatives, four hemoglobin stock solutions were prepared within 2–3 h after blood collection from the Quilcene hatchery following Riggs [5]. Red blood cells were pelleted by centrifugation (800 x g, 10 min, 4 °C), triple washed in ice cold 0.96% NaCl (aq) and resuspended to their original hematocrit in 20 mM phosphate buffered saline (PBS) at pH 8.0, and to 330 mOsm/kg using NaCl to approximate adult coho plasma osmolality [6]. Red cells were lysed by addition of phosphate-buffered 0.5% Triton X-100 and cellular debris was removed by centrifugation (10,000 x g, 12 min, 4 °C), before passing hemolysates through a 0.45 μm pore size polyethersulfone (PES) syringe filter. Coho hemoglobin stock solutions were stored at 4 °C for no longer than 4 days. Oxyhemoglobin and carboxyhemoglobin measurements were performed immediately following hemoglobin stock preparations to prevent autoxidation during storage time. Storage time of hemoglobin stocks did not affect measurements for the remaining hemoglobin derivatives.

### Quantification of total hemoglobin

To calculate molar absorptivities of coho hemoglobin derivatives, hemoglobin concentrations of the stock solutions were quantified on the basis of iron content [7] using inductively coupled mass-spectrometry (ICP-MS). Aliquots (1-mL) of hemoglobin stock solutions were digested in 3 mL of 50% nitric acid (v:v) and heated just under boiling temperature (75–80 °C) until the digestate was reduced to 1 ml and clear [8]. Samples were removed from heat, and an aliquot of 200 μl of 30% hydrogen peroxide was added to each cooled sample to ensure complete digestion of all remaining particulates, and heated again to remove the hydrogen peroxide. Digestates were prepared in triplicate and analyzed for their iron concentrations on a Perkin Elmer DRCe ICP-MS according to protocols described by EPA Standard Method 6020 [9].

### Spectroscopic measurements

Molar absorptivities of solutions containing hemoglobin derivatives were calculated from spectroscopic measurements following procedures adapted from Zijlstra et al. [2]. Coho hemoglobin solutions maintained at 1–4 °C were equilibrated to room temperature (20 °C) prior to obtaining absorbance measurements. Aliquots of coho hemoglobin stock solutions and 20 mM PBS (pH 8.0) were pipetted directly into 3.5-mL quartz cuvettes with pathlengths of 1 cm. Hemoglobin stock solutions were diluted with PBS to maintain an optical density below 2.0. Absorption spectra were obtained using a diode array HP 8453 UV–VIS diode array spectrophotometer across a wavelength range of 450–700 nm, with a 1-nm bandwidth and 2-second integration time.

### Preparation of oxyhemoglobin, carboxyhemoglobin, deoxyhemoglobin and hemichrome

The hemoglobin derivatives oxyhemoglobin (OHb), carboxyhemoglobin (COHb), and deoxyhemoglobin (HHb) were prepared by treatment with oxygen (O_2_) at ambient pressures, carbon monoxide (CO), and nitrogen (N_2_) gases, respectively [2]. N_2_ and CO gases were humidified with PBS prior to coho hemoglobin incubations under these gases. Coho oxyhemoglobin was prepared immediately following hemoglobin stock preparation by exposure to ambient oxygen partial pressures. Absence of methemoglobin contamination was confirmed by the lack of any observable peak in the ~630 nm spectral region, characteristic of methemoglobin. After obtaining the oxyhemoglobin spectra, the same samples were then incubated under humidified CO (g) for 15 min to form carboxyhemoglobin, and the carboxyhemoglobin spectra obtained. Deoxyhemoglobin was prepared by incubating the coho hemoglobin solutions on ice under N_2_ (g) until a single stable absorption peak was observed, which was achieved in 3–4 h. Trace oxygen contamination prevented the complete desaturation of coho hemoglobin, therefore N_2_ (g) was bubbled through a solution of vanadous chloride to completely remove oxygen before humidification with PBS [10].

Hemichrome (HemiHb) was prepared by diluting the coho hemoglobin stock solution in PBS containing 0.5 M sodium salicylate, followed by the addition of a slight molar excess of potassium ferricyanide [11]. A nearly complete conversion to hemichrome was achieved within 45 min, and then continued slowly up to about 4 h before changes in the spectral pattern were no longer discernable.

Coho oxyhemoglobin, deoxyhemoglobin, carboxyhemoglobin and hemichrome solutions were prepared in duplicate for each of four coho hemoglobin stock solutions.

### Preparation of methemoglobin

**Fig. 1:**
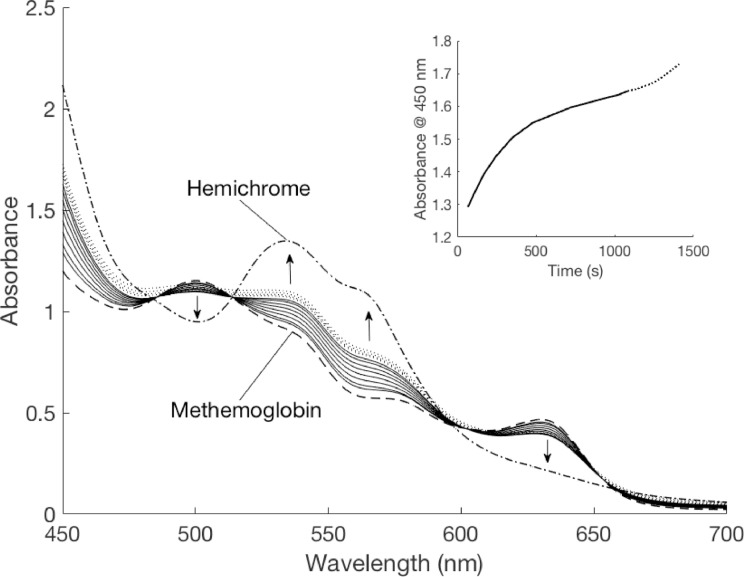
Absorbance spectral time series of coho hemoglobin solution after oxidation with a slight excess of potassium ferricyanide (solid lines). Arrows indicate the direction of peak changes. Modeled pure coho methemoglobin spectra (dashed lines) and pure hemichrome spectra (dashed-dotted lines) are overlaid. The spectral time series shows a shift in absorbance pattern from that most similar to pure methemoglobin, towards that of hemichrome over a period of around 18 min (inset) before the sample begins to precipitate (dotted lines), which is recognized as a rise in the baseline of the spectra and loss of isosbestic points.

The experimentally derived absorbance spectra of pure coho methemoglobin (MetHb) could not be obtained due to spontaneous hemichrome formation. Examination of the spectral time series of coho hemoglobin following the addition of ferricyanide revealed the complete conversion to methemoglobin within one minute, which was immediately and rapidly followed by hemichrome formation. Conversion of methemoglobin to hemichrome continued for about 15 min and subsequently led to precipitation (Fig. 1).

Following Alam and Callis [3], a model-based regression technique using multivariate statistics was applied in order to resolve the overlapping spectra of coho methemoglobin and hemichrome. The conversion of methemoglobin to hemichrome is considered to follow a pseudo-first order reaction in adult human hemoglobin [11], therefore a first order reaction was selected as the model for the applied chemical regression.

Given the time vector *n* (s), wavelength vector *m* (nm), a spectral data matrix with *m* x *n* dimensions, and the initial hemoglobin concentration (mM Fe), the objective was to determine the first order rate constant, ***κ***, that describes the conversion of methemoglobin to hemichrome:

(1) $$\text{MetHb}\overset{k}{\to }\text{HemiHb}$$

An iterative modeling method was applied using MatLab version R2017a as follows. The singular value decomposition of spectral data matrix **R** was determined, and then rank reduced to the first two eigenvectors to reduce noise.

(2) $$\bar{R}=\bar{\Sigma }\;\bar{U}\;{\bar{V}}^{T}$$

Variables in Eq. (2) represent the rank reduced factorization of the spectral data matrix, $\bar{R}\;,\;$with dimensions *m* x *n*. $\bar{V}$ is a *n* x 2 matrix that is proportional to concentrations of MetHb and HemiHb, and ${\bar{V}}^{T}$ is the transposed matrix of $\bar{V}$. $\bar{\Sigma }$ is a 2 × 2 matrix and $\bar{U}$ is a *m* x 2 matrix.

Based on first order kinetics, the integrated rate law in reaction (1) is expected to follow:

(3) $${\left[A\right]}_{t}={\left[A\right]}_{0}\;e^{-\kappa t}$$

Where **[A]_t_** is the concentration of methemoglobin at time ***t*** and **[A]_0_** is the initial concentration of methemoglobin. **[B]_t_** is the concentration of hemichrome at time ***t***, and is determined by the difference between the initial concentration of methemoglobin and the concentration of methemoglobin at time ***t***:

(4) $${\left[B\right]}_{t}={\left[A\right]}_{0}-{\left[A\right]}_{t}$$

The concentration matrix of **[A]_t_** and **[B]_t_** with *n* x 2 dimensions, termed **C**, can therefore be calculated based on the initial concentration of hemoglobin, as determined by ICP-MS analysis, when ***κ*** is known. As $\bar{V}$ is proportional to concentrations of methemoglobin and hemichrome through time, a rotation matrix **H** can be applied to the concentration matrix **C** to produce $\;{\bar{V}}^{T}$:

(5) $${\bar{V}}^{T}=\mathrm{HC}$$

A least-squares estimate of the rotation matrix, **Ĥ**, can be made based on an initial guess for ***κ***, ${\bar{V}}^{T}$ and the Moore-Penrose pseudoinverse [12] of the concentration matrix, **C^+^**:

(6) $$\hat{H}={\bar{V}}^{T}C^{+}$$

**Ĥ** can then be used to determine an estimate for ${\bar{V}}^{T}$, which is expressed as ${\overset{´}{V}}^{T}$:

(7) $${\overset{´}{V}}^{T}=\hat{H}C$$

After the initial guess for ***κ***, ${\overset{´}{V}}^{T}$ is then compared to ${\bar{V}}^{T}$. As iterative guesses are made for ***κ***, the **Ĥ, C**, ${\overset{´}{V}}^{T}$ matrices are recalculated. A Simplex optimization [13] was applied to determine the value of ***κ*** that minimized the difference between ${\overset{´}{V}}^{T}$ and ${\bar{V}}^{T}$.

Once a value for ***κ*** was determined, with concentration matrix **C**, a matrix **Ŕ** corresponding to the initial and final spectra of the chemical reaction (1) with *m* x 2 dimensions was calculated:

(8) $$\overset{´}{R}=\hat{H}\bar{\Sigma }{\bar{U}}^{T}$$

**Fig. 2:**
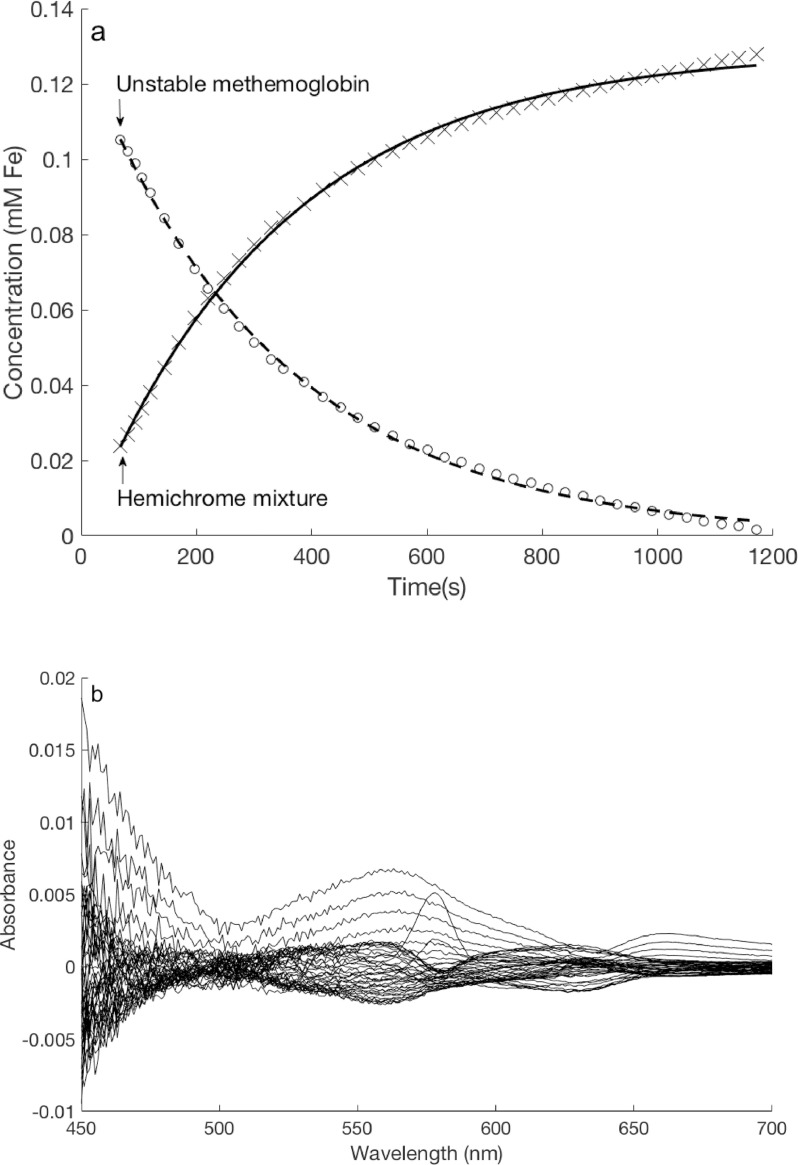
Chemical regression goodness-of-fit test results for one modeled spectral series. (A) The predicted relative decreasing concentrations of unstable coho methemoglobin (dashed line) and increasing concentrations of hemichrome (solid line) based on the first-order reaction model show close agreement with calculated concentrations (circles and x's) using the determined rotation matrix and time series absorption spectra. (B) Residuals between actual and predicted absorbance measurements throughout the conversion of coho methemoglobin to hemichrome are slight, affirming goodness-of-fit.

Using the rotation matrix **Ĥ**, relative concentrations of methemoglobin and hemichrome were determined for each of the absorbance spectra within the spectral data matrix **R** to check for agreement of the projected first order fit (Fig. 2A). Residuals between the actual absorbance and the predicted absorbance according to the first-order kinetics model were also used to verify goodness of fit (Fig. 2B).

The modeled spectra given in the **Ŕ** matrix were used to calculate the millimolar absorptivity of pure methemoglobin. Other outputs of the model-based chemical regression method were: (1) the pseudo-first order rate constant ***κ*** (s^−1^) of the partial conversion of methemoglobin to hemichrome; and (2) the final absorbance spectra of the reaction using MCA, which was used to calculate the fraction of hemoglobin within a sample that readily formed hemichrome upon oxidation. Coho methemoglobin solutions were prepared and analyzed in duplicate.

For methemoglobin molar absorptivity determination from pH range 7.8 to 6.8, solutions were prepared as prior using 20 mM PBS in 330 mOsmol/L using NaCl.

### Absorptivity calculation

Absorptivities were determined on the basis of the Beer-Lambert equation:

(9) $$ɛ=a{\left(c\;\iota \right)}^{-1}$$

Where **ε** is defined as the millimolar absorptivity constant (L mmol Fe^−1^ cm^−1^), **a** is the absorption spectrum of a solution containing a pure coho hemoglobin derivative, **c** is the hemoglobin concentration (mM Fe) and **ι** is the standard cuvette optical pathlength of 1 cm.

Due to the four or more hours required for deoxyhemoglobin preparation, a substantial amount of methemoglobin and hemichrome formed due to autoxidation. After obtaining the absorbance spectra of coho hemoglobin following incubation under nitrogen gas, deoxyhemoglobin was converted to carboxyhemoglobin by incubation under carbon monoxide gas. The contaminating concentrations of methemoglobin and hemichrome were determined using MCA and were used to correct deoxyhemoglobin absorbance spectra.

### Multi-component analysis calculation

The concentration of each hemoglobin derivative based on a 5-way multi-component analysis can be calculated in MatLab version R2017a, using the entire absorbance spectrum and a least-squares fit to the following formula based on the Beer-Lambert equation:

(10) $$a_{\mathrm{Hb}}=\iota \left({ɛ}_{\mathrm{OHb}}c_{\mathrm{OHb}}+{ɛ}_{\mathrm{HHb}}c_{\mathrm{HHb}}+{ɛ}_{\mathrm{COHb}}c_{\mathrm{COHb}}+{ɛ}_{\mathrm{MetHb}}c_{\mathrm{MetHb}}+{ɛ}_{\mathrm{HemiHb}}c_{\mathrm{HemiHb}}\right)$$

Given the 251 *×* 1 vector **a_Hb_**, corresponding to the absorbance spectrum (450–700 nm) of a coho hemoglobin sample containing a mixture of hemoglobin derivatives, and matrix **M_ε_**, with 251 × 5 dimensions corresponding to the molar absorptivities of the five coho hemoglobin derivatives, a least-squares fit of concentrations of each hemoglobin derivative **C**_Hb_ was found using **a_Hb_** and the Moore-Penrose pseudoinverse [12] of the molar absorptivity matrix **M_ε_^+^**.

(11) $$C_{\mathrm{Hb}}=M_{ɛ}^{+}a_{\mathrm{Hb}}$$

Multi-component analysis assumes that the model contains all hemoglobin derivatives present in the sample, as hemoglobin concentrations are estimated on the basis of a least-squares fit to the actual absorbance spectrum of a sample. Failure to include all pertinent hemoglobin derivatives into a model when calculating hemoglobin concentrations of a sample may lead to serious errors using multi-component analysis. Residual spectra may be helpful to identify the spectral absorbance pattern of any missing hemoglobin derivatives. Inclusion of unnecessary parameters in the model may lead to slightly negative concentrations for absent hemoglobin derivatives. Hemoglobin derivatives deemed absent from the sample should be removed from the model one at a time, and hemoglobin concentrations recalculated. Residuals of the final model in which all hemoglobin concentrations produce positive values should always be inspected visually for goodness-of-fit between the actual sample absorbance spectrum and the predicted spectrum as quality assurance prior to finalizing concentration results.

### Statistics

Results of coho millimolar absorptivity calculations are presented as mean ± standard error of the mean (SEM). Normality was verified using the Shapiro-Wilk's test. Differences between millimolar absorptivities experimentally determined for coho and previously published human hemoglobin at comparable pH values [14,^15^,2]] were tested for statistical significance (α= 0.01) using a two-tailed, one-sample Student's *t*-test in MatLab version R2017a. As the standard deviations of the published human values were unknown, a significance level of α = 0.01 was chosen instead of α = 0.05 to reduce the possibility of making a Type I error, and Bonferonni adjustments were applied for the determination of statistical significance using multiple comparisons. Wavelengths were selected corresponding to regions of peak maxima and minima between 450 and 700 nm where values were available for both species.

### Coho vs. human hemoglobin comparisons

**Fig. 3:**
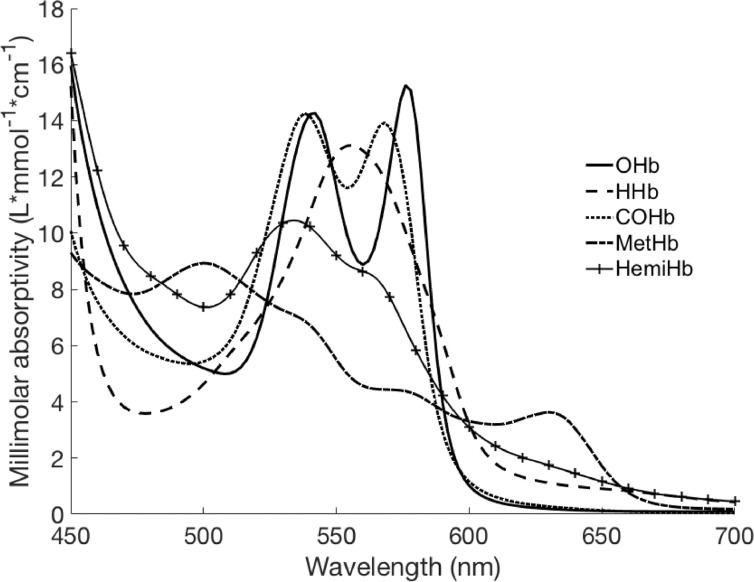
The millimolar absorptivities of major adult coho hemoglobin derivatives. Coho absorptivities are shown in the spectral range of 450–700 nm for oxyhemoglobin (OHb), deoxyhemoglobin (HHb), carboxyhemoglobin (COHb), methemoglobin (MetHb) and hemichrome (HemiHb) prepared in phosphate buffered saline at pH 8.0. Prespawn coho blood samples collected (*n* = 4) from the Quilcene hatchery (Quilcene, WA, USA).

Coho hemoglobin millimolar absorptivities derived in the current study (Fig. 3; Supplementary Table 1) demonstrated clear differences from those previously reported for humans (Table 1). Coho methemoglobin showed lower absorptivities in wavelengths near 540 nm and 576 nm and higher absorptivity at 630 nm when compared to human values [14] at pH 8.0 (Table 1). Coho and human carboxyhemoglobin spectra showed an overall similar pattern except for a lower peak maximum near 568 nm in coho. Oxyhemoglobin and deoxyhemoglobin spectral absorption patterns showed no differences between coho and human values published by Zijlstra et al. [2].

**Table 1:** Hemoglobin Millimolar Absorptivities of Adult Human and Prespawn Adult Coho.

λ (nm)	Coho (*n* = 4) (L mmol Fe^−1^ cm^−1^) Mean ± S.E.	Human (L mmol Hb^−1^ cm^−1^)	*P* Value
Oxyhemoglobin			
542	14.3 ± 0.07	14.52†	0.0262
560	8.88 ± 0.03	8.767†	0.0340
576	15.3 ± 0.09	15.26†	0.997
Deoxyhemoglobin			
476	3.60 ± 0.09	3.297†	0.0307
556	13.1 ± 0.08	13.36†	0.0455
Carboxyhemoglobin			
538	14.3 ± 0.06	14.30†	0.593
554	11.6 ± 0.05	11.63†	0.639
568	13.9 ± 0.06*	14.43†	2.50 × 10^−3^
Methemoglobin (pH 8.0)			
540	6.74 ± 0.06*	7.54‡	6.26 × 10^−4^
560	4.57 ± 0.06*	5.42‡	4.59 × 10^−4^
576	4.42 ± 0.05*	5.93‡	3.86 × 10^−5^
630	3.63 ± 0.05*	2.93‡	4.85 × 10^−4^
Hemichrome			
560	8.64 ± 0.05	8.6§	0.413
577	6.39 ± 0.02*	6.8§	3.17 × 10^−4^
630	1.74 ± 0.03*	0.92§	1.04 × 10^−4^

⁎ Indicates statistically significant difference between human and coho after Bonferroni corrections (*P* < 0.01);

† [2];

‡ [14];

§ [15].

### Methods validation

The accuracy of the coho MCA method was validated against a series of five standard solutions containing 4.7, 7.5, 9.7, 19.7 and 48.8% methemoglobin, prepared by mixing aliquots of 100% oxyhemoglobin and 100% methemoglobin stock solutions. Coho blood samples collected from the Skookumchuck hatchery were used to prepare oxyhemoglobin and methemoglobin stock solutions. A stock solution of 100% oxyhemoglobin was prepared from a coho blood sample collected the same day. A stock solution of coho methemoglobin was prepared by adding 18x molar excess of sodium nitrite to fully oxidize the hemoglobin. Sodium nitrite was used instead of potassium ferricyanide to minimize errors in hemoglobin determination of the stock solutions using ICP-MS on the basis of the iron content. To prevent uncontrolled hemoglobin oxidation in methemoglobin mixtures, residual nitrite was removed by injecting the methemoglobin stock solution into a 10 mL Pierce™ Slide-A-Lyzer (VWR) cassette and dialyzed against 1 L of 20 mM PBS and 4 exchanges of buffer. The methemoglobin stock solution was centrifuged (10,000 x g, 4 °C, 10 min) and filtered using a 0.45 μm pore size PES syringe filter. Based on previous experiments, it was expected that the prepared methemoglobin stock solution would consist of a mixture of coho methemoglobin and hemichrome. However, the coho methemoglobin in this stock solution did not lead to hemichrome formation, which was unexpected and not observed in any previous preparations of coho methemoglobin. Presently, it is unknown why coho methemoglobin instability may vary between individuals or populations, but one hypothesis may be differences in hemoglobin structures.

**Fig. 4:**
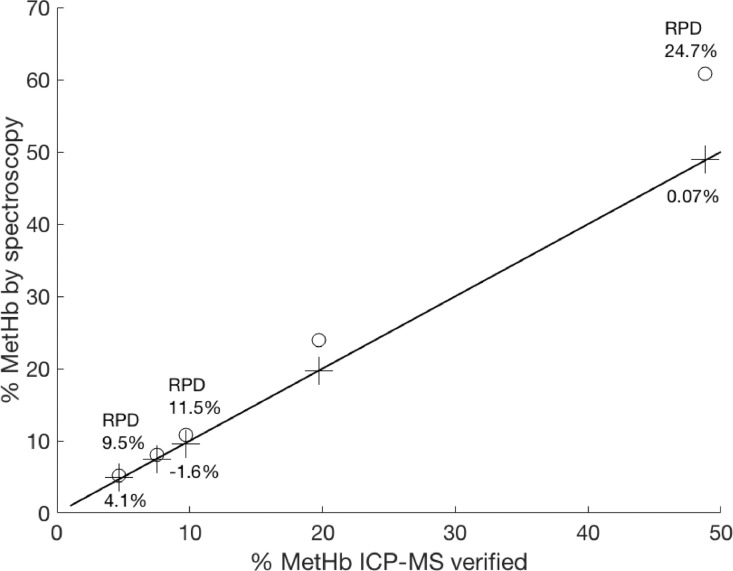
Comparison of percent coho methemoglobin results using the current coho multi-component analysis (MCA) method and a human multi-wavelength (MW) technique against a control method based on mixtures of 0% and 100% coho methemoglobin stock solutions, which were analyzed for iron content using ICP-MS. The coho MCA method (crosses) performed better than the human MW method (circles) following Benesch et al. [16] using human molar absorptivities published by van Assendelft and Zijlstra [14] to match assay pH conditions of 8.0. The solid line shows a 1:1 relationship between the control and spectroscopic methods. Relative percent differences (RPD) are shown for the Benesch et al. [16] MW method above the line, and for the coho MCA method below the line. The adult coho blood sample was collected from the Skookumchuck hatchery (Tenino, WA, USA).

Fig. 4 shows the correlation between the control method (ICP-MS verified percent methemoglobin) and two spectroscopic methods: the current coho hemoglobin MCA method and the multi-wavelength method described by Benesch et al. [16] using the corrected human molar absorptivities reported by van Assendelft and Zijlstra [14]. Acceptance criterion of methemoglobin results using either the coho MCA or human multi-wavelength method was set at a relative percent difference of 5% of values determined using ICP-MS.

Concentrations of methemoglobin calculated using MCA all fell within 5% of predicted values based on mixtures of 100% coho oxyhemoglobin and 100% methemoglobin stock solutions. In contrast, the multi-wavelength method consistently overestimated the methemoglobin levels of the coho hemoglobin mixtures (Fig. 4) and did not meet the quality assurance criteria in any of the five control methemoglobin solutions. At low methemoglobin percentages (<5%), both methods performed reasonably well, with a percent difference <10% relative to controls (Fig. 4). However, the multi-wavelength method does not account for the presence of unstable methemoglobins that would be immediately evident with the current MCA approach. Since spontaneous hemichrome formation may vary between species, populations and individuals, it is difficult to predict the presence of unstable methemoglobins prior to sample analysis. Above 10% methemoglobin, the multi-wavelength method increasingly overestimated the results, with percent differences 11–25% relative to controls. In contrast, the coho MCA results approximated a 1:1 relationship with the control method.

### Interspecific hemichrome formation in Pacific salmon

To explore hemoglobin instability in several species of Pacific salmon, blood samples were collected from Chinook (*n* = 4), pink (*n* = 2), chum (*n* = 2) and steelhead (*n* = 4) to determine the rate constants and fractions of hemichrome-forming methemoglobins. Collected samples were stored at 1–4 °C and analyzed the same day using the chemical regression method described above. Hemoglobin solutions were diluted to an optical density below 2.0 using isotonic 20 mM PBS or 10 mM Tris saline solution at pH 8.0. Assays were performed at 20 °C until precipitation was observed or for at least 30 min if no precipitation was observed in the hemoglobin solution.

The average rate constants for hemichrome formation in adult coho, Chinook, pink and chum methemoglobin were 0.0030 ± 0.0002 *s* ^−^ ^1^, 0.0024 ± 0.0004 *s* ^−^ ^1^, 0.0022 ± 0.0003 *s* ^−^ ^1^, 0.0033 ± 0.0002 *s* ^−^ ^1^, respectively. No hemichrome formation was detected in samples of adult steelhead methemoglobin. Unstable methemoglobin is defined as the fraction of total methemoglobin that spontaneously forms hemichrome during assay conditions. Because the final spectra of the reactions contained a mixture of methemoglobin and hemichrome, not all fish methemoglobins appear to spontaneously form hemichrome. The percentage of unstable methemoglobin that spontaneously formed hemichrome for coho, Chinook, pink and chum was 42 ± 5%, 50 ± 6%, 44 ± 6%, 30 ± 5%, respectively.

Because hemichrome formation was not observed for steelhead, the cyanide-derivative method may be appropriate for this and other fish species possessing stable methemoglobin using the neutralized cyanide reagent recommended by Lacey and Rodnick [17]. The cyanide-derivative method may not be appropriate for species that display immediate and spontaneous hemichrome formation, as was observed in coho, Chinook, pink and chum salmon.

The stable methemoglobin observed in the coho blood sample collected from the Skookumchuk hatchery for coho MCA method validation was unexpected. Although sodium nitrite rather than potassium ferricyanide was used as the oxidizing agent for this sample, stable coho methemoglobin oxidized with potassium ferricyanide was demonstrated in two additional adult coho also sampled from the Skookumchuck hatchery (data not shown), therefore the type of chemical oxidant does not seem to influence methemoglobin stability. Furthermore, we observed considerable hemichrome formation during the autoxidation of coho hemoglobin solutions sampled from the Quilcene hatchery and Chinook deoxyhemoglobin samples, absent any added chemical oxidizing agents (Supplementary Table 3). The presence of coho and Chinook hemichrome following partial autoxidation supports evidence that methemoglobin instability may be a property of hemoglobin structure [18].

### Method considerations

The reversible binding of oxygen by hemoglobin requires red blood cells to maintain the heme iron in its reduced ferrous (Fe^2+^) state. As a transition metal, heme iron is readily oxidized to its ferric (Fe^3+^) form, forming methemoglobin, which does not bind oxygen. Methemoglobin is not only a product but also a mediator of oxidative stress in red blood cells, as it is effective at catalyzing the formation of free radicals and reactive oxygen/nitrogen species (ROS/RNS) in the presence of oxygen and oxidizing/reducing agents [19]. Fishes may be more susceptible to complications associated with methemoglobin formation due to a greater intrinsic pro-oxidative potential of their hemoglobins compared to their avian and mammalian counterparts [21,20]. Given the essential role of fish hemoglobins in both oxygen transport and mediation of intraerythrocytic ROS/RNS production, a simple and reliable method for fish methemoglobin determination is a useful tool for monitoring fish health where water quality degradation is a concern.

Routine optical methods in methemoglobin determination take advantage of the unique methemoglobin absorption band at 630 nm and follow one of two general approaches. The first is based on the change in absorbance at 630 nm following conversion of methemoglobin to cyanmethemoglobin. This method was first used by Evelyn and Malloy [22] who added acid-neutralized cyanide to divided hemoglobin solutions in order to relate the change in absorbance at 630 nm before and after complete oxidation using potassium ferricyanide. The second approach utilizes the Beer-Lambert relationship between concentration and the unique light absorbance pattern of hemoglobin derivatives (e.g. oxyhemoglobin, deoxyhemoglobin, methemoglobin, etc.), which requires the prior acquisition of molar absorptivity datasets for each hemoglobin derivative at each wavelength in question [23]. Benesch et al. [16] simplified the process by publishing a series of multi-wavelength equations to quantify methemoglobin based on light absorption of human red blood cell lysates at just a few wavelengths. Commercially available co-oximeters further facilitated the practice of clinical routine methemoglobin analysis based on multi-wavelength optical methods that incorporated turbidity corrections for use with whole blood samples.

While the Evelyn and Malloy [22] method remains a gold standard in accuracy and precision for methemoglobin determination in mammals, several studies have found this approach to be unsuitable for use in fishes. For example, unexplained precipitation occurred using the cyanide-derivative method with the hemoglobin samples of many tropical marine fish species examined by Wells et al. [24]. Similarly, Root [25] reported a ‘coagulum’ formed by the addition of potassium ferricyanide to some fish hemoglobin solutions. Hesser [26] concluded that the cyanide-derivative method is not suitable for use in fish species where this precipitation interference is observed.

The internal binding of ferric (Fe^3+^) heme to an amino acid ligand along the globin chain gives rise to soluble and insoluble hemichromes [4]. Although concentrations of hemichromes are often very low in human hemoglobins, they form readily under denaturing conditions and in unstable hemoglobin variants where abnormalities in the amino-acid structure, particularly of the heme pocket, facilitate the spontaneous conversion of methemoglobin to hemichrome [27]. Insoluble hemichromes form precipitation products known as Heinz bodies, which can be visually identified in red blood cells supravitally stained with methyl violet 2B or new methylene blue [28]. Therefore, it cannot be assumed that human and fish hemoglobins behave similarly under all assay conditions and special consideration must be given to methodological artifacts that may arise in the determination of methemoglobin in fishes. For fishes with unstable methemoglobins, multi-component analysis (MCA) using spectroscopic absorptivity of each hemoglobin derivative is required to accurately determine the relative abundance of hemoglobin derivatives.

Calculation errors in methemoglobin abundance results if species-specific differences in molar absorptivities are not corrected for in MCA [29,30]. Inter-specific variation in methemoglobin absorptivity is expected because the ferric (Fe^3+^) heme site of methemoglobin is liganded by either a water molecule or by a hydroxide ion, the relative ratios of which are influenced by the configuration of the heme pocket and pH [31]. The liganded state of the heme iron determines the absorptivity pattern of methemoglobin, which also depends on the overall hemoglobin structure and pH [29,32]. Although oxyhemoglobin and carboxyhemoglobin are also slightly modified by pH [33], [34], the effect is negligible for most quantitative purposes [32]. Fishes possess a multiplicity of hemoglobin structures, with very distinct functional and structural features compared to mammals [35], therefore notable distinctions from mammals in their methemoglobin molar absorptivities should not be surprising.

Hemoglobin quantification techniques are fundamental to the routine evaluation of fish health [36,37]. Techniques first developed for use in humans have had long-standing use in fish studies with a potential for complications due to inherent differences in fish and human hemoglobin characteristics, such as hemoglobin instability. In the current study, the instability of coho, Chinook, pink and chum salmon methemoglobins interfered with accurate methemoglobin quantification and we adapted a spectroscopic multi-component analysis approach to improve upon previous available methods. Notably, Heinz bodies appeared prominently in red blood cells of even apparently healthy coho, in our facility as well as various independent regional hatcheries, which would be expected in a species that possesses unstable methemoglobin [18]. To our knowledge, this study is the first report of the inherent instability of methemoglobin in Pacific salmon (*Oncorhynchus spp*.). This approach may be particularly useful for investigating the toxicological significance of methemoglobin formation in fishes in the context of exposure to environmental contaminants and other co-occurring stressors, which may be an under-appreciated contributor to early mortality in Pacific salmon.

## CRediT authorship contribution statement

**Stephanie Blair:** Conceptualization, Methodology, Validation, Formal analysis, Investigation, Data curation, Writing - original draft, Visualization, Supervision, Project administration, Funding acquisition. **Clyde Barlow:** Conceptualization, Methodology, Software, Formal analysis, Resources, Writing - review & editing. **Erin Martin:** Conceptualization, Writing - review & editing, Supervision. **Ruth Schumaker:** Investigation, Validation, Writing - review & editing. **Jenifer McIntyre:** Conceptualization, Resources, Writing - review & editing, Visualization, Funding acquisition.

## Appendix Supplementary materials

Table S1. Molar absorptivities (L mmol Fe^−1^ cm^−1^) of coho oxyhemoglobin (OHb), deoxyhemoglobin (HHb), methemoglobin (MetHb) and hemichrome (HemiHb) in the wavelength range of 450–700 nm. Image, application 1

Table S2. Molar absorptivities (L mmol Fe^−1^ cm^−1^) of coho methemoglobin (MetHb) pH 7.8-6.8 Image, application 2

Table S3. Coho and Chinook methemoglobin and hemichrome formation by autoxidation during incubation under nitrogen gas. Image, application 3

## References

[1] Scholz N.L., Myers M.S., McCarthy S.G., Labenia J.S., McIntyre J.K., Ylitalo G.M., Rhodes L.D., Laetz C.A., Stehr C.M., French B.L., McMillan B., Wilson D., Reed L., Lynch K., Damm S., Davis J., McMillan B. (2011). Recurrent die-offs of adult coho salmon returning to spawn in puget sound lowland urban streams. PLoS ONE.

[2] Zijlstra W.G., Buursma A., van Assendelft O.W. (2000). Visible and Near Infrared Absorption Spectra of Human and Animal hemoglobin: Determination and Application.

[3] Alam M.K., Callis J.B. (1994). Elucidation of species in alcohol-water mixtures using near-IR spectroscopy and multivariate statistics. Anal. Chem..

[4] Rachmilewitz E.A. (1969). Formation of hemichromes from oxidized hemoglobin subunits. Ann. N. Y. Acad. Sci..

[5] Riggs A. (1981). Preparation of blood hemoglobins of vertebrates.

[6] Donaldson M.R., Clark T.D., Hinch S.G., Cooke S.J., Patterson D.A., Gale M.K., ..., Farrell A.P. (2010). Physiological responses of free-swimming adult coho salmon to simulated predator and fisheries encounters. Physiol. Biochem. Zool..

[7] Zijlstra W.G., Van Kampen E.J. (1960). Standardization of hemoglobinometry: I. The extinction coefficient of hemiglobincyanide at λ= 540 mμ: ε540HiCN. Clin. Chim. Acta.

[8] Tyman R.M. (2005). Wet digestion. Anal. Chem..

[9] U.S. Environmental Protection Agency (1990). Method 6020 CLP-M: Inductively Coupled Plasma Mass Spectrometry.Washington DC: USEPA. Available atwww.epa.gov/sites/proINTRODUCTION. Electronic References:

[10] Atha D.H., Ackers G.K. (1974). Calorimetric determination of the heat of oxygenation of human hemoglobin as a function of pH and the extent of reaction. Biochemistry.

[11] Rachmilewitz E.A., Peisach J., Blumberg W.E. (1971). Studies on the stability of oxyhemoglobin a and its constituent chains and their derivatives. J. Biol. Chem..

[12] Penrose R. (1956). On best approximate solutions of linear matrix equations. Math. Proc. Camb. Philos. Soc..

[13] Nelder J.A., Mead R. (1965). A simplex method for function minimization. Comput. J..

[14] Van Assendelft O.W., Zijlstra W.G. (1975). Extinction coefficients for use in equations for the spectrophotometric analysis of hemoglobin mixtures. Anal. Biochem..

[15] Winterbourn C.C. (1985). Free-radical production and oxidative reactions of hemoglobin. Environ. Health Perspect..

[16] Benesch R.E., Benesch R., Yung S. (1973). Equations for the spectrophotometric analysis of hemoglobin mixtures. Anal. Biochem..

[17] Lacey J.A., Rodnick K.J. (2002). Important considerations for methemoglobin measurement in fish blood: assay choice and storage conditions. J. Fish Biol..

[18] Winterbourn C.C. (1990). Oxidative denaturation in congenital hemolytic anemias: the unstable hemoglobins. Semin. Hematol..

[19] Nohl H., Stolze K. (1998). The effects of xenobiotics on erythrocytes. Gen. Pharmacol. Vasc. Syst..

[20] Richards M.P., Dettmann M.A. (2003). Comparative analysis of different hemoglobins: autoxidation, reaction with peroxide, and lipid oxidation. J. Agric. Food Chem..

[21] Aranda R., Cai H., Worley C.E., Levin E.J., Li R., Olson J.S., Phillips G.N., Richards M.P. (2009). Structural analysis of fish versus mammalian hemoglobins: effect of the heme pocket environment on autooxidation and hemin loss. Proteins Struct. Funct. Bioinform..

[22] Evelyn K.A., Malloy H.T. (1938). Microdetermination of oxyhemoglobin, methemoglobin, and sulfhemoglobin in a single sample of blood. J. Biol. Chem..

[23] Van Kampen E.J., Zijlstra W.G. (1966). Determination of hemoglobin and its derivatives.

[24] Wells R.M., Baldwin J., Seymour R.S. (1997). Low concentrations of methemoglobin in marine fishes of the great barrier reef, Australia. Mar. Freshw. Res..

[25] Root R.W. (1931). The respiratory function of the blood of marine fishes. Biol. Bull..

[26] Hesser E.F. (1960). Methods for routine fish hematology. Progress. Fish Cult..

[27] Rifkind J.M., Abugo O., Levy A., Heim J. (1994). Detection, formation, and relevance of hemichromes and hemochromes.

[28] Bain B.J., Bates I., Laffan M.A. (2016). Dacie and Lewis Practical Hematology.

[29] Zijlstra W.G., Buursma A. (1987). Spectrophotometry of hemoglobin: a comparison of dog and man. Comp. Biochem. Physiol. B.

[30] Zijlstra W.G., Buursma A. (1997). Spectrophotometry of haemoglobin: absorption spectra of bovine oxyhemoglobin, deoxyhemoglobin, carboxyhemoglobin, and methemoglobin. Comp. Biochem. Physiol. Part B Biochem. Mol. Biol..

[31] Di Iorio E.E., Antonini E., Rossi-Bernardi L., Chicone E. (1981). Preparation of derivatives of ferrous and ferric hemoglobin.

[32] Zijlstra W.G., Buursma A., Meeuwsen-Van der Roest W.P. (1991). Absorption spectra of human fetal and adult oxyhemoglobin, de-oxyhaemoglobin, carboxyhemoglobin, and methemoglobin. Clin. Chem..

[33] Wimberley P.D., Fogh-Andersen N., Siggaard-Andersen O., Lundsgaard F.C., Zijlstra W.G. (1988). Effect of pH on the absorption spectrum of human oxyhemoglobin: a potential source of error in measuring the oxygen saturation of hemoglobin. Clin. Chem..

[34] Wimberley P.D., Siggaard-Andersen O., Fogh-Andersen N. (1990). Accurate measurements of hemoglobin oxygen saturation, and fractions of carboxyhemoglobin and methemoglobin in fetal blood using radiometer OSM3: corrections for fetal hemoglobin fraction and pH. Scand. J. Clin. Lab. Invest..

[35] Jensen F.B., Fago A., Weber R.E. (1998). Hemoglobin structure and function. Fish Physiol..

[36] Blaxhall P.C., Daisley K.W. (1973). Routine hematological methods for use with fish blood. J. Fish Biol..

[37] Roche H., Bogé G. (1996). Fish blood parameters as a potential tool for identification of stress caused by environmental factors and chemical intoxication. Mar. Environ. Res..

