# Supplementary material for: Methemoglobin determination by multi-component analysis in coho salmon (Oncorhynchus kisutch) possessing unstable hemoglobin
Source: MethodsX. 2020 Feb 22;7:100836. doi: 10.1016/j.mex.2020.100836 (PMC7115134; doi:10.1016/j.mex.2020.100836)
Supplement: Supplementary file 3 — Table S3. Coho and Chinook methemoglobin and hemichrome formation by autoxidation during incubation under nitrogen gas. [file mmc3.docx]

| **Table S3. Coho and Chinook Methemoglobin and Hemichrome Formation by Autoxidation** | | |
| --- | --- | --- |
| Sample^†^ (n=4) | Percent Methemoglobin  (Mean ± S.D.) | Percent Hemichrome  (Mean ± S.D.) |
| *Coho* | | |
| 1 | 2.2 ± 0.2 | 6.2 ± 0 |
| 2 | 1.4 ± 0 | 4.5 ± 0.1 |
| 3 | 2.5 ± 0.4 | 5.5 ± 0.1 |
| 4 | 2.2 ± 0 | 4.5 ± 0.1 |
| *Chinook* | | |
| 1 | 1.0 ± 0.1 | 2.4 ± 0.07 |
| 2 | 8.7 ± 0.2 | 16 ± 0.01 |
| 3 | 2.0 ± 1.0 | 7.7 ± 0.04 |
| 4 | 3.0 ± 0.4 | 9.4 ± 0.03 |
| ^†^ Individual samples prepared in duplicate. Coho and Chinook sampled from Quilcene (Quilcene, WA) and George Adams (Shelton, WA) fish hatcheries, respectively. | | |
